# Supplementary figures and images for: TNFα Activates the Liver X Receptor Signaling Pathway and Promotes Cholesterol Efflux from Human Brain Pericytes Independently of ABCA1
Source: Int J Mol Sci. 2023 Mar 22;24(6):5992. doi: 10.3390/ijms24065992 (PMC10056409; doi:10.3390/ijms24065992)

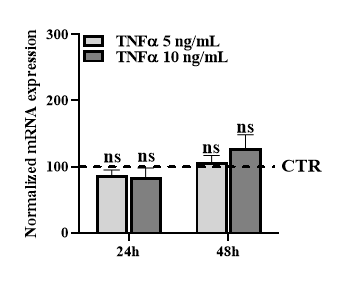

Supplement: Supplementary file 1 [file ijms-24-05992-s001.zip › Supplementary Figure S1.TIF]

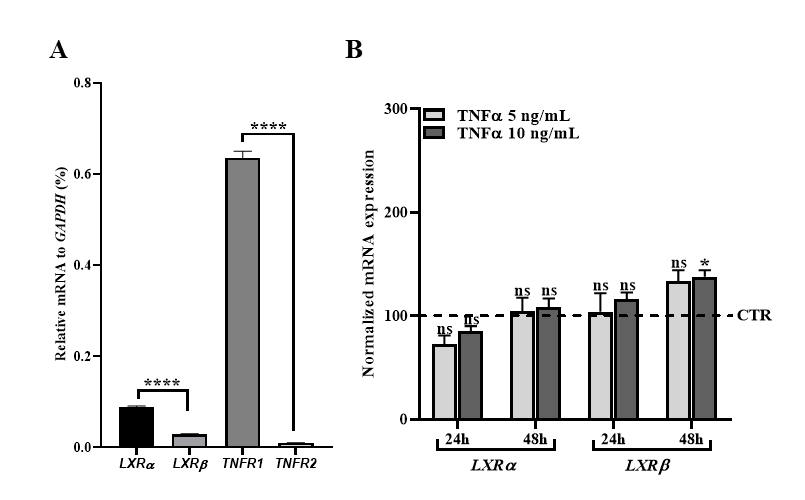

Supplement: Supplementary file 1 [file ijms-24-05992-s001.zip › Supplementary Figure S2.tif]

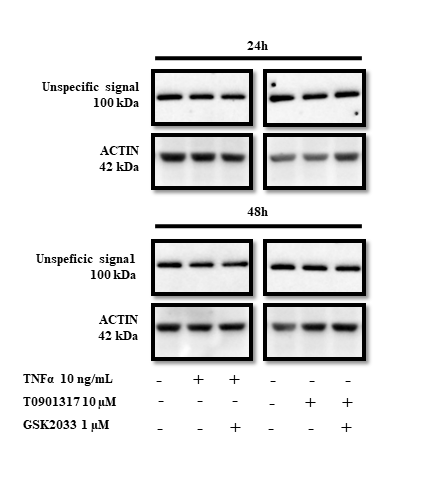

Supplement: Supplementary file 1 [file ijms-24-05992-s001.zip › Supplementary Figure S3.TIF]

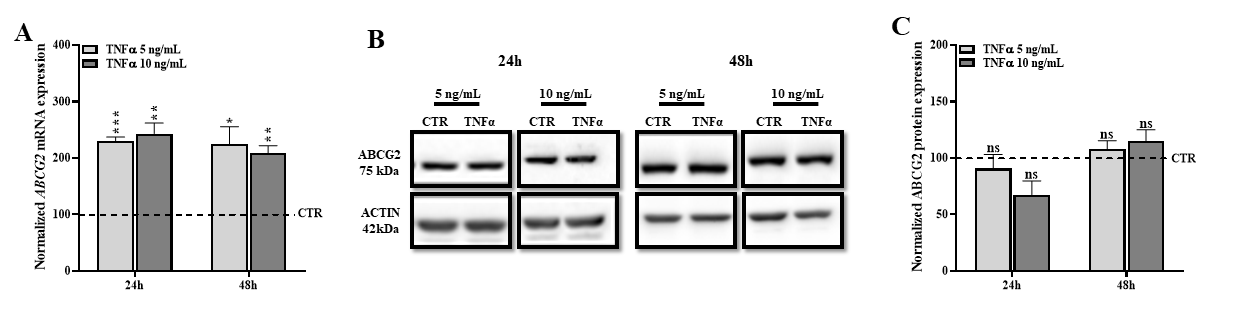

Supplement: Supplementary file 1 [file ijms-24-05992-s001.zip › Supplementary Figure S4.TIF]

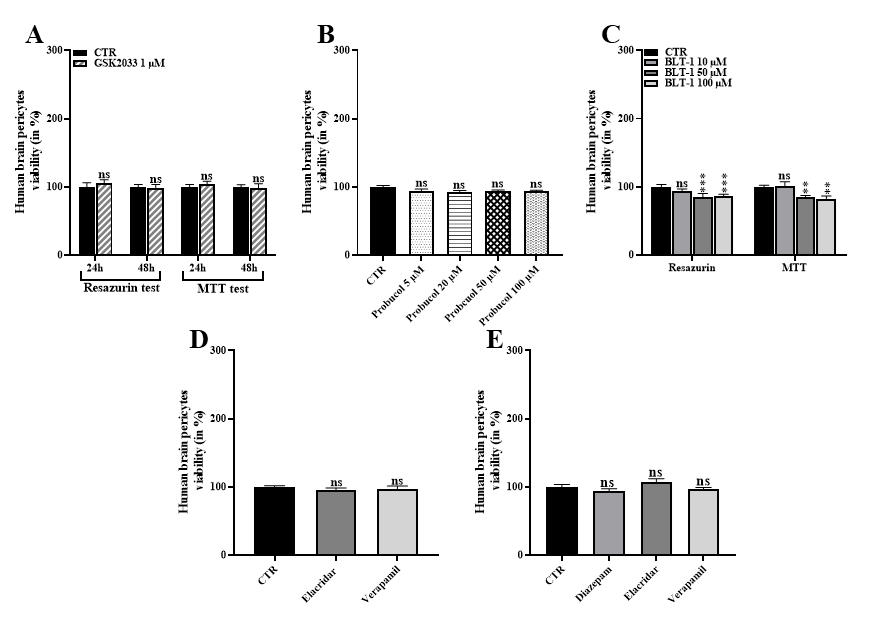

Supplement: Supplementary file 1 [file ijms-24-05992-s001.zip › Supplementary Figure S5.TIF]

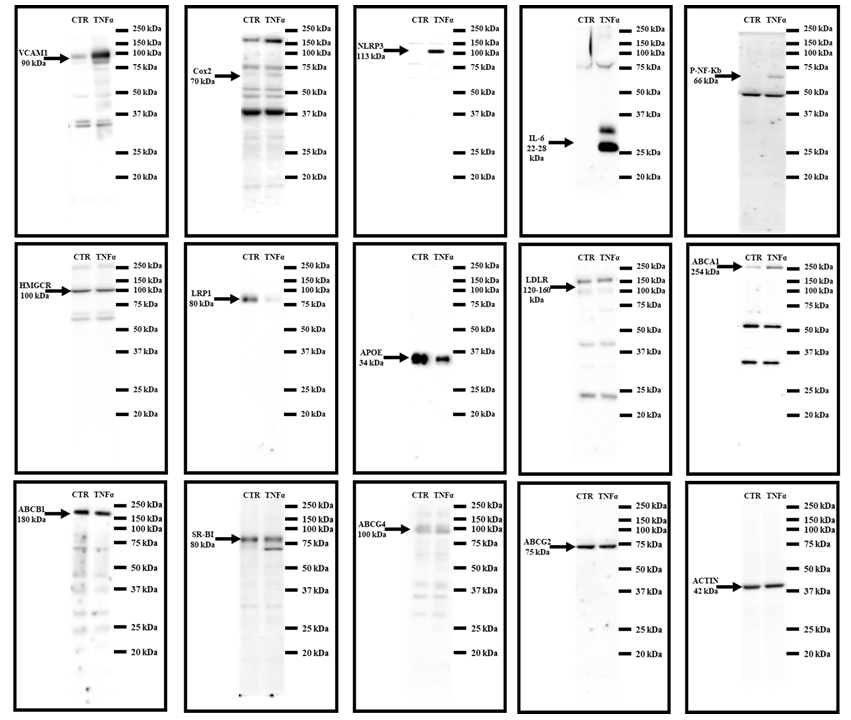

Supplement: Supplementary file 1 [file ijms-24-05992-s001.zip › Supplementary Figure S6.TIF]
